# Tick–Host–Pathogen Interactions: Conflict and Cooperation

**DOI:** 10.1371/journal.ppat.1005488

**Published:** 2016-04-21

**Authors:** José de la Fuente, Margarita Villar, Alejandro Cabezas-Cruz, Agustín Estrada-Peña, Nieves Ayllón, Pilar Alberdi

**Affiliations:** 1 SaBio, Instituto de Investigación en Recursos Cinegéticos IREC-CSIC-UCLM-JCCM, Ciudad Real, Spain; 2 Department of Veterinary Pathobiology, Center for Veterinary Health Sciences, Oklahoma State University, Stillwater, Oklahoma, United States of America; 3 Center for Infection and Immunity of Lille (CIIL), INSERM U1019–CNRS UMR 8204, Université Lille Nord de France, Institut Pasteur de Lille, Lille, France; 4 Facultad de Veterinaria, Universidad de Zaragoza, Zaragoza, Spain; University of North Carolina at Chapel Hill School of Medicine, UNITED STATES

## Tick-Borne Pathogens: The Model

Ticks are blood-feeding arthropod ectoparasites that transmit pathogens that constitute a growing burden for human and animal health worldwide [1–3]. Only second to mosquitoes as vector of human diseases and the first vector of animal diseases, ticks transmit bacterial, parasitic, and viral pathogens [1]. One of these pathogens is the intracellular bacterium *Anaplasma phagocytophilum*, which is vectored primarily by *Ixodes* tick species and is the causative agent of human granulocytic anaplasmosis (HGA), equine and canine granulocytic anaplasmosis, and tick-borne fever of ruminants [1]. This pathogen is a good model because recent analysis of the molecular interactions between *Ixodes* tick vectors, *A*. *phagocytophilum*, and host cells showed pathogenic effects of both ticks and pathogens but also revealed the mutual beneficial effects of the tick–host–pathogen molecular interactions [4–7].

## Tick–Host–Pathogen Interactions: Conflict and Cooperation

**Fig 1:**
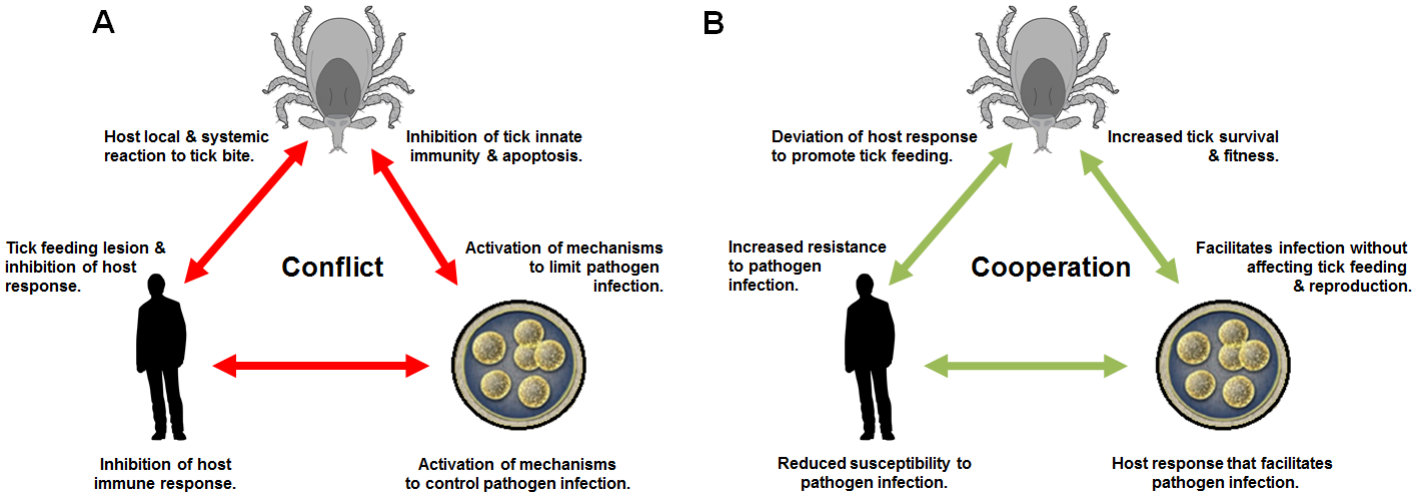
Tick–host–pathogen interactions: conflict and cooperation. (A) Conflict. Ticks produce a feeding lesion and inhibit host hemostatic, immune, and inflammatory responses to complete feeding, while hosts react locally and systemically to tick infestation. Ticks react to pathogen infection by activating different mechanisms to limit pathogen infection, while pathogens manipulate tick biological processes such as innate immune response and apoptosis to facilitate infection, multiplication, and transmission. Pathogens inhibit host immune response, among other mechanisms, to facilitate infection, but at the same time, hosts react to pathogen infection by activating different mechanisms to control pathogen infection. (B) Cooperation. Ticks benefit from hosts by promoting feeding after deviation of host response to tick bite, while hosts may benefit from tick infestation by increased resistance to pathogen infection. Ticks benefit from pathogen infection by increased survival at low and high temperatures and fitness, while pathogens manipulate tick biological processes to facilitate infection but without affecting tick feeding and reproduction. Pathogens benefit from host response to facilitate infection, while hosts may benefit from pathogen infection by interference with and reduced susceptibility to infection with other more lethal pathogens or by bacterial-induced epigenetic deregulations that could promote host defense to infection.

It has been established that ticks produce a feeding lesion and inhibit host hemostatic, immune, and inflammatory responses to complete feeding, while pathogens manipulate host and tick biological processes to facilitate infection, multiplication, and transmission [4–7]. At the same time, both ticks and hosts react to tick infestation and/or pathogen infection by activating different mechanisms to fight against tick infestations and limit pathogen infection [4–7]. Therefore, the generally accepted view is that tick infestation and pathogen infection produce detrimental effects on both hosts and ticks that highlight a conflict between hosts, ticks, and pathogens (Fig 1A; see also S1 Video) [5,7]. The evolutionary processes show that coevolution includes interactions between organisms that can produce both conflict and cooperation [8], but the latter has been largely ignored for tick–host–pathogen interactions. However, the conflict between ticks, hosts, and pathogens also reveals cooperation between them benefiting ticks and pathogens and to a lesser extent hosts, leading to mutual beneficial effects of the tick–host–pathogen molecular interactions (Fig 1B; see also S1 Video). The conflict and cooperation in tick–host–pathogen interactions are analyzed in detail in the following sections with examples summarized in Table 1.

**Table 1:** Examples of the conflict and cooperation events acting on tick–host–pathogen interactions.

Interactions	Affected	Conflict	Benefit and/or Cooperation
Tick–pathogen	Tick	Porin down-regulation to inhibit host intrinsic apoptosis pathway [9].	Induction of tick antifreeze glycoprotein (AFGP) and heat shock proteins (HSP) [11,12].
	Pathogen	FAS down-regulation to activate host extrinsic apoptosis pathway [9]. Dual oxidase activation to induce host production of reactive oxygen species (ROS) [10].	Promotion of tick protein misfolding in the endoplasmic reticulum (ER) [13].
Host–tick	Host	Down-regulation of lectin and complement activation [7,16].	Increased antibody levels to α-gal [21–23].
	Tick	Activation of host coagulation and platelet aggregation.	Increased tick feeding after the effect of tick saliva on host immunity and inflammatory responses [4,6,7,16].
Host–pathogen	Host	Inflammatory histopathologic lesions and neutropenia [17].	Bacterial-induced epigenetic deregulations and production of host interleukin (IL)-10 [17,18,26].
	Pathogen	Host production of pathogen-specific immunoglobulin G (IgG) and CD4-dependent inflammatory responses [18].	Increased levels of host IL-8, CXCR1, and other chemokines [19,20].

The conflict affects tick, pathogen, or host biology and/or life cycle, while benefit and/or cooperation results in beneficial effects to increase tick, pathogen, or host fitness.

### (a) Tick–pathogen interaction: conflict for both ticks and pathogens

Like other intracellular bacteria, *A*. *phagocytophilum* have evolved mechanisms to subvert host response to facilitate infection, multiplication, and transmission [5]. These molecular mechanisms for infection of tick cells include but are not limited to remodeling of the cytoskeleton, inhibition of cell apoptosis, manipulation of the immune response, and control of host cell epigenetics [5]. For example, in tick salivary glands, *A*. *phagocytophilum* inhibits the intrinsic apoptosis pathway through porin down-regulation to facilitate bacterial infection, while tick cells respond through FAS down-regulation, resulting in the activation of the extrinsic apoptosis pathway to limit *A*. *phagocytophilum* infection and promote tick survival (Table 1) [9]. Alterations in the tick gut microbiome associated with feeding, development, and infection could modulate immune response in ticks [10]. Microbiota and *A*. *phagocytophilum*-induced activation of dual oxidase results in production of reactive oxygen species (ROS) to control bacteria and activate immune responses as well as epithelial regeneration and repair to protect ticks from infection (Table 1) [10]. However, ROS-mediated damage to gut epithelial cells results in activation of the Janus kinase/signal transducers and activators of transcription (JAK/STAT) pathway, which in turn inhibits apoptosis that facilitates infection of tick salivary glands [9,10].

### (b) Tick–pathogen interaction: benefits for both ticks and pathogens

**Fig 2:**
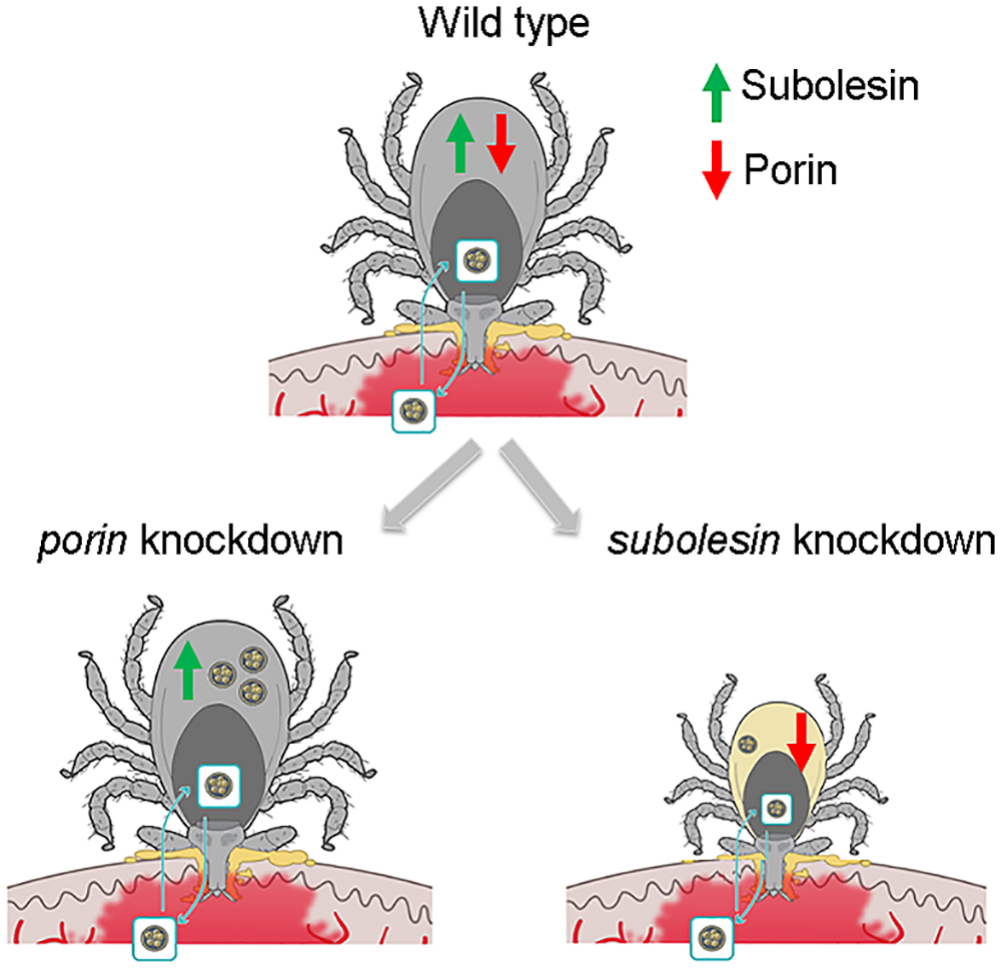
*Ixodes scapularis* tick–*A*. *phagocytophilum* coevolution. The pathogen inhibits apoptosis by reducing porin levels to increase infection but without affecting tick feeding and reproduction, as illustrated after gene knockdown to maintain tick vector capacity. However, the pathogens do not manipulate subolesin levels because, as shown after gene knockdown, it can affect infection and tick performance. These results illustrate coevolutionary mechanisms by which pathogens manipulate tick protective responses to facilitate infection while preserving tick feeding and vector capacity to guarantee survival of both pathogens and ticks.

At the tick–pathogen interface, pathogens induce several mechanisms to increase tick survival and favor pathogen infection and transmission. These mechanisms include the induction of an antifreeze glycoprotein (AFGP) and heat shock proteins (HSP) (Table 1) [11,12]. The AFGP increases tick survival at cold temperatures [11], while the HSP response helps to increase tick survival by protecting from stress and preventing desiccation at high temperatures after enhancing questing speed in order to increase chances to attach to a host [12]. In addition, because pathogen infection occurs during blood feeding, ticks have developed a protective response to limit pathogen infection, which also contributes to their survival [12–14]. *A*. *phagocytophilum* subvert tick RNA interference by mechanisms other than reducing tudor staphylococcal nuclease (Tudor-SN) levels to preserve tick life cycle because of the role of this protein during tick feeding [14]. In contrast, subolesin, which is involved in tick innate immune response to limit pathogen infection [15], is not manipulated by *A*. *phagocytophilum* infection because it affects tick feeding and reproduction and infection with tick-borne bacteria (Fig 2) [15].

On the pathogen side, *A*. *phagocytophilum* could promote protein misfolding in the endoplasmic reticulum (ER) to counteract the tick cell response to infection, but tick cells respond by activating protein targeting and degradation to prevent ER stress and cell apoptosis, a mechanism that facilitates pathogen infection (Table 1) [13]. Additionally, *A*. *phagocytophilum* may benefit from the tick cell ability to limit rickettsial infection through phosphoenolpyruvate carboxykinase (PEPCK) inhibition, leading to decreased glucose metabolism and the availability of essential metabolites for bacterial growth, which also results in the inhibition of cell apoptosis that increases infection of tick cells [13].

### (c) Host–tick and host–pathogen interactions: conflict for hosts, ticks, and pathogens

Tick infestations produce a feeding lesion and inhibit host cell responses such as immunity by down-regulation of lectin, complement activation, and other mechanisms to impair the activity of natural killer cells, neutrophils, eosinophils, basophils, and T lymphocytes (Table 1) [7,16]. In turn, hosts respond to tick infestation by activating different mechanisms, including coagulation and platelet aggregation, that affect tick feeding and blood digestion (Table 1) [7,16]. Pathogens manipulate host biological processes to facilitate infection, multiplication, and transmission [4–7]. For example, *A*. *phagocytophilum* infection results in inflammatory histopathologic lesions and neutropenia (Table 1) [17]. However, host response to *A*. *phagocytophilum* infection produces pathogen-specific IgG against bacterial major surface proteins and CD4-dependent inflammatory responses of activated macrophages and neutrophils to control pathogen infection (Table 1) [18].

### (d) Host–tick and host–pathogen interactions: benefits for ticks and pathogens

The mechanisms described above, by which tick saliva modulates host immunity and suppresses inflammatory responses, deviate the host immune response to facilitate tick feeding and pathogen transmission (Table 1) [4,6,7,16].

The mechanisms by which *A*. *phagocytophilum* subvert host response to facilitate infection, multiplication, and transmission appear to be common to tick vectors and vertebrate hosts, suggesting an evolutionary adaptation to a diverse number of vector and host species [5]. Therefore, these coevolutionary mechanisms are also reflected at the host–pathogen interface, where neutrophils infected with *A*. *phagocytophilum* show up-regulation of proinflammatory genes and increased levels of interleukin 8 (IL-8), IL-8 receptor (CXCR1), and other chemokines [19,20]. These protective responses result in the recruitment of neutrophils and increased granulocytic phagocytosis, which in turn facilitate *A*. *phagocytophilum* dissemination (Table 1) [20].

### (e) Host–tick and host–pathogen interactions: benefits for the host

A remaining question is what is the benefit for the host from both ticks and pathogens? One hypothesis is that hosts may benefit from the capacity of ticks to manipulate their immune response [16]. For example, in humans, tick feeding may result in increased antibody levels to the carbohydrate α-gal (Gal α 1-3Gal β 1-[3]4GlcNAc-R) [21,22]. Although the tick-induced response to α-gal may result in anaphylactic reactions to red meat, tick bite, and cetuximab treatment, it could also increase protection to pathogen infection [21–23]. Likely, most tick-borne pathogens contain α-gal-modified proteins on their surface. Therefore, increased antibody levels to α-gal could contribute to reducing pathogen infection and multiplication (Table 1) [21–23].

It is difficult to consider that pathogen infection may have a benefit for the host. However, reservoir hosts and not accidental hosts that do not play a role during tick and pathogen life cycle can control pathogen infection to guarantee survival and facilitate pathogen transmission. These facts suggest the existence of mechanisms that evolved to produce a beneficial effect after pathogen infection. Pathogen interference may constitute one of these mechanisms in which pathogen infection, as shown for tick microbiota [10], may interfere with and reduce the susceptibility to infection with other more lethal pathogens [24]. Another possible mechanism is the epigenetic modification produced by *A*. *phagocytophilum* that affects the chromatin structure and transcriptional program of host cells [25]. Bacterial-induced epigenetic deregulations may affect host cell function, resulting in pathogen persistence but also promoting host defense to infection (Table 1) [26]. Additionally, the induction of IL-10 in response to *A*. *phagocytophilum* infection results in the control of histopathologic lesions induced by host-derived interferon gamma (IFN-γ) (Table 1) [17,18].

## Conclusions and Future Directions

The evolution of the tick–host–pathogen molecular interactions resulted in conflict and cooperation between them, with mutual beneficial effects for ticks, hosts, and pathogens (see S1 Video). These results illustrate coevolutionary mechanisms by which pathogens manipulate tick protective responses to facilitate infection while preserving tick feeding and vector capacity to guarantee survival of both pathogens and ticks (Fig 2). The conflict between hosts, ticks, and pathogens has been well characterized. However, the beneficial effects are being discovered for ticks and pathogens and require additional research to provide more evidence for their presence in vertebrate hosts. As discussed here for ticks and *A*. *phagocytophilum*, these coevolutionary mechanisms probably apply to other arthropod vectors and transmitted pathogens.

Because of the growing impact of tick-borne pathogens on human and animal health, more effective measures are needed for the control of tick-borne diseases, and the understanding of the molecular interactions between vertebrate hosts, tick vectors, and transmitted pathogens is crucial towards achieving this goal [15]. The characterization of the conflict and mutual beneficial effects of the tick–host–pathogen molecular interactions will likely provide new targets for the control of tick-borne diseases. The possibility of ticks inducing cross-reactive protective antibodies to α-gal that could increase protection to pathogen infection opens new research areas to control and prevent vector-borne diseases [21,22].

## Supporting Information

S1 Video Tick–host–pathogen interactions: conflict and cooperation. (M4V)
